# Trends in Hospital Admission and Surgical Procedures Following ED visits for Diverticulitis

**DOI:** 10.5811/westjem.2016.4.29757

**Published:** 2016-06-13

**Authors:** Margaret B. Greenwood-Ericksen, Joaquim M. Havens, Jiemin Ma, Joel S. Weissman, Jeremiah D. Schuur

**Affiliations:** * Brigham and Women’s Hospital, Department of Emergency Medicine, Boston, Massachusetts; † Massachusetts General Hospital, Department of Emergency Medicine, Boston, Massachusetts; ‡ Harvard Medical School, Boston, Massachusetts; § Brigham and Women’s Hospital, Center for Surgery and Public Health, Department of Surgery, Boston, Massachusetts; ¶ Brigham and Women’s Hospital, Division of Trauma, Burns and Surgical Critical Care, Boston, Massachusetts; || Surveillance and Health Services Research Program, American Cancer Society, Atlanta, Georgia

## Abstract

**Introduction:**

Diverticulitis is a common diagnosis in the emergency department (ED). Outpatient management of diverticulitis is safe in selected patients, yet the rates of admission and surgical procedures following ED visits for diverticulitis are unknown, as are the predictive patient characteristics. Our goal is to describe trends in admission and surgical procedures following ED visits for diverticulitis, and to determine which patient characteristics predict admission.

**Methods:**

*:* We performed a cross-sectional descriptive analysis using data on ED visits from 2006–2011 to determine change in admission and surgical patterns over time. The Nationwide Emergency Department Sample database, a nationally representative administrative claims dataset, was used to analyze ED visits for diverticulitis. We included patients with a principal diagnosis of diverticulitis (ICD-9 codes 562.11, 562.13). We analyzed the rate of admission and surgery in all admitted patients and in low-risk patients, defined as age <50 with no comorbidities (Elixhauser). We used hierarchical multivariate logistic regression to identify patient characteristics associated with admission for diverticulitis.

**Results:**

Fryom 2006 to 2011 ED visits for diverticulitis increased by 21.3% from 238,248 to 302,612, while the admission rate decreased from 55.7% to 48.5% (−7.2%, 95% CI [−7.78 to −6.62]; p<0.001 for trend). The admission rate among low-risk patients decreased from 35.2% in 2006 to 26.8% in 2011 (−8.4%, 95% CI [−9.6 to −7.2]; p<0.001 for trend). Admission for diverticulitis was independently associated with male gender, comorbid illnesses, higher income and commercial health insurance. The surgical rate decreased from 6.5% in 2006 to 4.7% in 2011 (−1.8%, 95% CI [−2.1 to −1.5]; p<0.001 for trend), and among low-risk patients decreased from 4.0% to 2.2% (−1.8%, 95% CI [−4.5 to −1.7]; p<0.001 for trend).

**Conclusion:**

From 2006 to 2011 ED visits for diverticulitis increased, while ED admission rates and surgical rates declined, with comorbidity, sociodemographic factors predicting hospitalization. Future work should focus on determining if these differences reflect increased disease prevalence, increased diagnosis, or changes in management.

## INTRODUCTION

Colonic diverticular disease is increasingly prevalent in the developed world and affects more than half of the population over the age of 65 years.^1^ It is estimated that approximately 20% of patients with diverticulosis develop diverticulitis over the course of their lifetime.^2^ Diverticulitis frequently causes abdominal pain, which accounts for approximately 8% of U.S. emergency department (ED) visits.^3^ Approximately 300,000 patients are admitted to U.S. hospitals for diverticulitis each year, accounting for 1.5 million days of inpatient care per year.^4^,^5^

Treatment of diverticulitis is based on comorbidities and severity, with severe disease requiring admission and possible surgical intervention.^6^,^7^ A recent meta-analysis^8^ and prospective randomized control trial^9^ both demonstrate the safety of outpatient management with oral antibiotics for uncomplicated diverticulitis. In 2014 the American Society of Colon and Rectal Surgeons recommended outpatient management in selected patients with uncomplicated diverticulitis.^7^,^10^ Despite evidence to support outpatient management, the published literature has reported increased admission and surgical rates from the late 1990s to early 2000s.^11^,^12^

With the increasing prevalence of diverticular disease and the increasing role of the ED in management of acute conditions, we aimed to determine if there has been a change in hospital admission and surgery among ED patients with diverticulitis. This study analyzed data from a national all-payer hospital billing dataset to evaluate the prevalence, the rate of admission, and the rate of surgical intervention for patients with diverticulitis who presented to the ED. Additionally, we determined patient predictors of admission for patients. Specifically, we hypothesized that rates of admission and surgery have decreased in recent years.

## METHODS

### Study Design and Data Source

We conducted a cross-sectional descriptive analysis using data on ED visits from 2006–2011 to determine change in admission and surgical patterns over time. Additionally, to determine patient predictors of admission, we performed a multiple variable logistic regression analysis, adjusting for patient comorbidity using the system developed by Elixhauser.^13^ This study was approved by the institutional review board at Brigham and Women’s Hospital.

The Nationwide Emergency Department Sample (NEDS) was used for our analysis. NEDS is a U.S. administrative database that is part of the Healthcare Cost and Utilization Project.^14^ NEDS is a component of the Healthcare Cost and Utilization Project (HCUP) of the Agency for Healthcare Research and Quality (AHRQ); it is the largest all-payer ED database publicly available in the U.S.^14^ NEDS contains 26 to 29 million ED records per year from approximately 950 annually selected hospitals, which represents roughly a 20% stratified sample of hospital-based EDs in the U.S.^14^

NEDS uses a complex sampling design stratified by sampling weight, geographic region, trauma center designation, urban–rural status, teaching hospital status, and hospital ownership to allow for calculation of national estimates.^14^ Visit details available in NEDS include patient demographics, visit disposition (home, transfer to another facility, admitted to hospital, or expired), and up to 15 diagnoses from the final location (e.g. inpatient diagnoses are from the hospital bill while diagnoses for patients discharged from the ED are from the ED bill). By incorporating sampling weights provided in NEDS, we were able to generate national estimates for ED utilization at both hospital and visit level in the U.S. More detailed descriptions of NEDS can be found elsewhere.^14^

### Study Population

We included ED visits by adult patients, 18 years and older, who had an International Classification of Diseases, Ninth Revision, Clinical Modification (ICD-9-CM) code for diverticulitis of the large colon (562.11, 562.13) as their principal diagnosis. In sensitivity analyses, we included ED visits where diverticulitis was a secondary diagnosis and where the principal diagnosis was thought to be diverticulitis-related, e.g. abdominal pain (Appendix A).

We excluded patients with a disposition of neither discharge nor admission (left against medical advice, not admitted, destination unknown, or died in the ED; 0.38%). We also excluded hospitals with <10 cases (18.8% of hospitals; 1.1% of visits) because low hospital volumes result in unstable estimates of admission rates. We excluded patients with complicated diverticulitis as defined by the American College of Surgeons^7^ (i.e., peritonitis, obstruction, perforation and abscess) and those with sepsis or shock, because virtually all such patients should be admitted to the hospital from the ED (Appendix B). We defined low-risk patients as those with no Elixhauser comorbidities and as age less than 50, which is defined as “young” by the American College of Colon and Rectal Surgeons.^7^

### Study Outcome and Variables

The primary outcome of interest was hospital admission after ED visits. We classified patients as admitted if they were admitted to the hospital or transferred to an acute care hospital after the initial ED visit, because the decision to transfer a patient represents a similar use of hospital care rather than discharging the patient to outpatient management. Patients were classified as discharged if their disposition was “routine ED discharge,” “transfer to skilled nursing or intermediate care facility,” “home health care,” or “discharge or transfer to court or law enforcement.”

An additional outcome of interest was the rate of surgical procedures; the surgical rate was calculated for all admitted patients and the low-risk sub-group. Data for outpatient, elective surgery were not available. We defined surgery as patients with at least one ICD-9 procedure code that indicated the patient had undergone a colectomy (45.7× or 45.94), a low anterior resection (48.6x), a colostomy (46.1x), an ileostomy (46.2x), a laparotomy (54.11 or 54.19), diagnostic laparoscopy (54.21), laparascopic lysis of adhesions (54.51), or percutaneous drainage (54.91).

### Data Analysis

National estimates of ED visits, admission rates and surgical rates for diverticulitis were estimated accounting for NEDS’s complex sampling design and sampling weights. We tested the trend in admission and surgical rates from 2006 – 2011 by logistic regression modeling by calendar year. The admission rate was defined as the number of patients admitted or transferred to another hospital, divided by the number of ED visits. The surgical rate was defined as the number of patients who underwent a surgical procedure, divided by the number of ED visits. Additionally, we determined an inpatient surgical rate in all admitted patients and in low-risk, admitted patients. The inpatient surgical rate was defined as the number of patients who underwent a surgical procedure, divided by the number of admitted patients. As our study population is a subset of NEDS, we applied subset analysis methods as recommended by AHRQ to obtain correct variance estimates for these descriptive statistics.

Patient predictors include age at time of visit, gender, insurance status (private, Medicare, Medicaid, self-pay/no charge, and other), median household income (quartile within the patient’s home ZIP code), and comorbid illness. We adjusted for comorbity using the system developed by Elixhauser. For each ED visit, we created dummy variables for each comorbidity cluster defined by Elixhauser, based on secondary diagnosis codes 13 and also created three dummy variables for additional conditions identified as likely to increase the chance of admission for diverticulitis that are not included in Elixhauser (“GI symptom,” “GI disease,” “disease severity”). For example, leukocytosis and acute renal failure are examples of diagnoses grouped under “disease severity,” that would increase a patient’s risk of being admitted with a principal diagnosis of diverticulitis, while benign prostatic hypertrophy is not. To determine these diagnoses, one author (MBG-E) reviewed all secondary codes on patients admitted with diverticulitis and flagged those that would increase the likelihood of admission. Independently, a surgical expert (JMH) reviewed the codes, and disagreements were resolved by discussion (Appendix C).

### Statistical Analyses

We report descriptive statistics and compare trends across years using chi-square tests for trend. To account for patient clustering within EDs and the associated clustering of care patterns for admission and surgery, we created hierarchical multivariate logistic regression models using validated analytical methods used by Centers for Medicare and Medicaid Services for analyzing administrative claims to determine morbidity and readmission.^15^ The models included patient and hospital characteristics as covariates. As suggested by the HCUP, sampling weights were not used in multilevel modeling. All analyses were done in SAS 9.3 (SAS, Cary, NC).

## RESULTS

**Figure 1:**
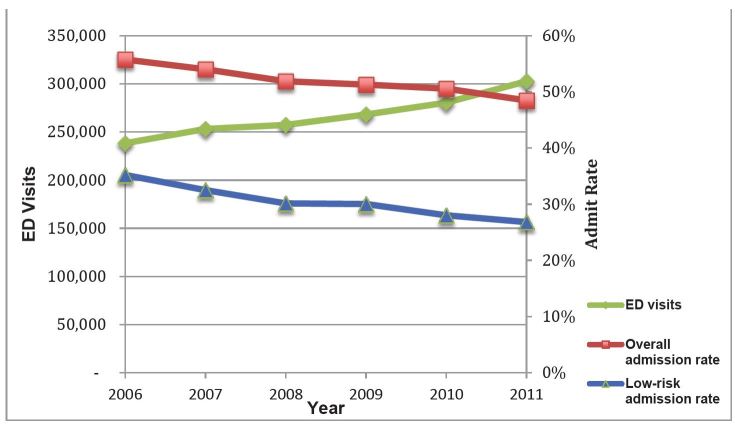
National trend in emergency department (ED) diagnosis and admission rates for diverticulitis, 2006–2011. *See Table 2 for values. For all trends, p<0.001.

In 2011 there were 302,612 ED visits for diverticulitis. Mean patient age was 58 years, the majority were female (56.7%), with the plurality having private insurance (43.7%), presenting to metropolitan non-teaching hospitals (50.1%), and being located in the southern region of the U.S. (41%; Table 1). ED visits increased by 21.1% from 2006 to 2011 (Figure 1). From 2006 to 2011, admission rates decreased from 55.7% to 48.5% (−7.2%, 95% CI [−7.78 to −6.62]; test for trend, p<0.001 (Figure 1 and Table 2).

**Figure 2:**
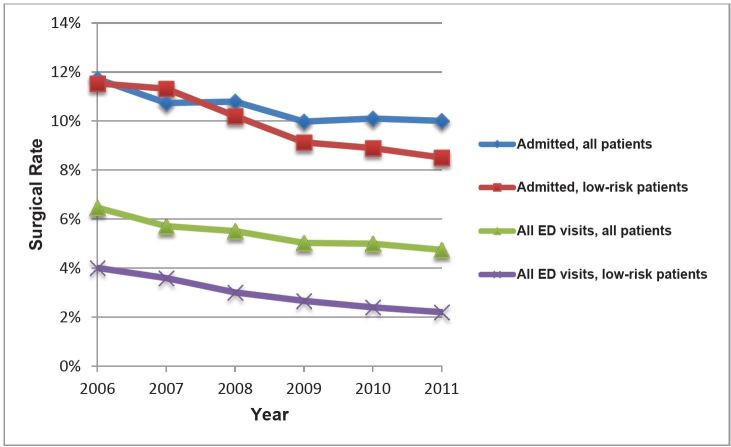
National trend in rates of surgery for diverticulitis, 2006–2011. *ED,* emergency department *See Table 3 for values. For all trends, p<0.001.

The rate of surgery decreased from 6.5% in 2006 to 4.7% in 2011 (−1.8%, 95% CI [−2.1 to −1.5]; test for trend, p<0.001) for all patients after an ED visit for diverticulitis. Among patients admitted to the hospital, the rate of surgery decreased from 11.7% in 2006 to 10.0% in 2011 (−0.7%, 95% CI [−1.2, −0.2]; p<0.001 for trend; Figure 2 and Table 3).

From 2006 to 2011, the admission rates for low risk patients decreased from 35.2% to 26.8% (−8.4%, 95% CI [−9.7 to −7.1]; test for trend, p<0.001; Figure 1 and Table 2). Among all low-risk patients, the rate of surgery decreased from 4% in 2006 to 2.2% in 2011 (−1.8%, 95% CI [−2.3 to −1.3]; test for trend, p<0.001; Figure 2 and Table 3) and among low-risk, admitted patients the surgery rate decreased from 11.5% in 2011 to 8.5% in 2006 (−3%, 95% CI [−4.5 to −1.5]; p<0.001 for trend; Figure 2 and Table 3).

The likelihood of admission varied by patient’s clinical and socioeconomic characteristics (Table 4). After adjustment, increased age was not associated with increased likelihood of admission, while male gender (OR of 1.19 with 95% CI [1.13 to 1.23]) was. All Elixhauser comorbidity factors were associated with significantly increased likelihood of admission (Appendix D), except congestive heart failure (OR of 1.08 with 95% CI [0.93 to 1.25]), AIDS (OR of 1.64 with 95% CI [0.62 to 4.35]) and uncomplicated diabetes mellitus (OR of 1.03 with 95% CI [0.97 to 1.10]). Medicare and Medicaid insurance types had a decreased likelihood of admission when compared to private insurance. Other non-private insurance types (e.g., Veterans’ Affairs) were associated with increased likelihood of admission. Self-pay was not associated with ED admission. Highest quartile income was associated with increased likelihood of admission when compared to lowest quartile.

## DISCUSSION

We analyzed a large U.S. all-payer hospital claims dataset to determine recent trends in the rates of admission and rates of surgery for diverticulitis after ED visits, and assessed how patient characteristics affect admission and surgery. We found that while ED visits for diverticulitis increased by about a fifth from 2006–2011, admission rates and rates of surgical intervention declined. We identified important comorbidities and sociodemographic factors predicting hospitalization.

The rising incidence of ED visits for diverticulitis may be related to an increasing prevalence of diverticular disease, changes in care-seeking patterns, or changes in diagnostic behavior. The aging population, low dietary fiber intake, and rising rates of obesity all contribute to a rising prevalence of diverticular disease in the U.S.^16^,^17^ This could result in more episodes of acute diverticular disease and more ED visits. As the ED has become the rapid diagnostic center of the U.S. health system, patients are more likely to get acute, unscheduled care in an ED rather than at a primary care physician’s or specialist’s office.^18^ These changes likely are true for diverticulitis – with patients more likely to be referred to an ED initially and to receive the diagnosis of diverticulitis at the ED rather than at a primary care physician’s or surgeon’s office.

Finally, the increased availability, use and resolution of computed tomography (CT) have likely increased the diagnosis of diverticulitis, whereas before clinical features would have led to the diagnosis. Changes in referral patterns and increasing use of CT have likely shifted the spectrum of disease, meaning that on average ED patients diagnosed with diverticulitis have less severe cases.^11^,^12^,^16^,^17^ Our findings are in parallel with two earlier studies based on the national inpatient sample that also found increased incidence of diverticulitis.^11^,^12^ However, these studies, performed 10 years ago, found increased rates of admission and surgical procedures over time, contrary to our findings. Further research is needed to determine whether the change to less admission and surgery is due to changes in disease incidence or patterns of medical care.

The declining rate of overall and among low-risk patients reflects changes in the ED population with diverticulitis and changes to the surgical guidelines. Recent research and treatment recommendations, including the American Society of Colon and Rectal Surgeons’ practice parameters, support outpatient management for uncomplicated diverticulitis and first attempting medical, rather than surgical management, for those admitted with diverticulitis.^7^,^19^,^20^ Our findings suggest that the surgical community is changing its standard of care towards non-operative management and increased outpatient management for diverticulitis. It is unlikely that the change in management is completely explained by a shifting spectrum of disease, with lower severity cases of diverticulitis being diagnosed in the ED. The move to less surgery and more outpatient management is patient-centered on face, as few patients want surgery or to be hospitalized. Additionally, low rates of operative management raise questions on the necessity of CT for clinical diverticulitis in younger, low-risk patients. Future research should aim to determine if the relationship between declining admission and surgical rates are related to changes in disease incidence, practice patterns or severity. Analysis of large datasets merged with electronic health record clinical data could demonstrate if severity is changing, by evaluation of clinical data including vital signs, radiology results, and lab tests to identify evidence of sepsis, abscesses or perforation.

We defined age<50 in our definition of low risk, as the American Society of Colorectal Surgeons recommends against routine elective resection in younger patients (<50 years).^7^ Of note, management of young patients (less than 50 years of age) with diverticulitis is one area of controversy. The controversy arose from several papers from the 1990s that reported a more severe course of disease and higher complication rates in young patients,^21^–^24^ while more recent studies and meta-analysis found no difference from disease behavior in older age groups.^19^,^25^–^30^ Current analysis suggests that the prior studies were performed before the CT era and included only a small number of patients, putting them at risk of misclassification and selection bias due to recognition of more severe cases and exclusion of mild cases.^25^ This group, similar to other age groups, has experienced an increase in rates of diagnosis,^11^,^12^ which is likely related to increased rates of diverticulitis. The obesity epidemic and dietary preferences are associated with increased prevalence of diverticular disease. Additionally, increased use of CT in the young, and increased awareness of diverticulitis as a potential diagnosis in the this age group have likely led to more frequent diagnosis.^31^,^32^

Our data demonstrate a decrease in the rates of admission and surgery in ED patient visits for diverticulitis from 2006–2011, with low-risk patients having lower admission and surgical rates when compared to the overall population. This indicates these decreased rates are likely due to changes in practice pattern, though it is possible that decreased virulence of diverticulitis is playing a role. It is further notable that surgical procedures declined despite including percutaneous drainage. This was included as a surgical procedure as it indicates an intervention and would require admission. Further investigation should be done to determine if lower admission rates in low-income patients result in worse clinical outcomes as evidenced by return visits or complications. We also evaluated the rates of surgical procedure in admitted patients, and again compared the rates overall to the rates in the low-risk population. The percentage of surgery is similar for admitted low-risk patients (10.5%) when compared to all admitted patients (10.0%) with overlapping CIs. This suggests that once admitted, the primary factor affecting the decision to operate is illness severity, rather than age and comorbid conditions.

We found that patients with Medicare and Medicaid were less likely to be admitted than privately insured patients after adjustment for patient factors and comorbid conditions. The data on the effect of insurance status and the decision to admit patients with diverticulitis from the ED are mixed. One study found higher rates of “avoidable” admissions in uninsured and Medicaid patients,^33^ and several other studies found lower rates of admission for uninsured and underinsured patients.^34^–^36^ We found a similar pattern between patient’s income and admission: patients residing in areas with the highest quartile of income were more likely to be admitted. As we do not have associated quality or outcomes data, in this analysis we cannot determine if this represents a quality issue – if wealthier, privately insured patients are being admitted too often or if lower-income, non-privately insured patients are being admitted too infrequently.

## LIMITATIONS

Our analysis has several limitations. Administrative claims datasets are susceptible to coding errors or misclassification of the diagnosis and disposition. However, as these records are used in hospital billing, there are regulatory standards and financial incentives to have correct diagnoses and dispositions. In the NEDS, patients are not uniquely identified, so they may account for multiple visits by the same patient within the sample; similarly, we are unable to determine if readmission from an ED visit was related to recent hospital discharge for the same condition. While this happens, for diverticulitis it likely represents a small proportion of the sample. Administrative data does not include clinical data regarding the severity of illness at the time of initial ED presentation, which makes it difficult to determine if management is driven by practice change or change in disease severity. Our data analysis was risk adjusted with the Elixhauser index, a well validated predictor of in-hospital mortality, and additional conditions unique to diverticulitis were added by study authors after discussion and consensus. Yet, no co-morbidity index completely controls for all co-morbid conditions. NEDS does not include observation care, which is an increasingly used pathway for the management of certain conditions,^37^ though as of 2011, diverticulitis has been an infrequent condition treated in observation.^37^ NEDS includes only ED visits; therefore, our analysis is limited to patients admitted to the hospital through the ED; we cannot comment on overall hospital admission rates.

## CONCLUSION

From 2006 to 2011, ED visits for diverticulitis increased while the admission rates and surgical rates decreased. The same trend was found in low-risk patients. Admission rates for diverticulitis are associated with various patient factors, with a trend towards increased admission rates for privately insured and wealthier patients. Despite increases in incidence, admission and surgery rates for younger, healthier patients decreased from 2006 to 2011. These results are in alignment with recent studies and guidelines supporting outpatient treatment for healthy patients with diverticulitis. On face, the reductions in admission, surgery and hospitalization appear patient oriented. However, variation in rates of admission surrounding socioeconomic status raise questions about disparities in care, and more research should be done to better understand if there have been changes in patient outcomes or disparities as practice patterns have changed.

## Supplementary Information









## Figures and Tables

**Table 1:** Patient and hospital characteristics for diverticulitis emergency department visits, 2011.

Characteristics	N	%
Mean age (SD)	66,656	57.6 (0.06)
Female	37,760	56.7
Insurance		
Medicare	23,264	35.0
Medicaid	5,568	8.4
Private insurance	29,078	43.7
Self-pay/no charge	6,585	9.9
Other	2,017	3.0
Income		
Lowest quartile	15,490	23.7
Second quartile	15,843	24.2
Third quartile	17,433	26.7
Highest quartile	16,604	25.4
Region		
Northeast	13,442	20.2
Midwest	12,755	19.1
South	27,331	41.0
West	13,128	19.7
Teaching status		
Metropolitan, non-teaching	33,400	50.1
Metropolitan, teaching	22,655	34.0
Non-metropolitan	10,601	15.9
Emergency department volume		
<20,000	7,087	10.6
20,000–49,999	26,591	39.9
≤50,000	32,978	49.5

*Income*, quartile of the median household income of the patient’s home ZIP code.

*Region*, as defined by the U.S. Census Bureau.

**Table 2:** National estimates of emergency department (ED) volume and admission rate for diverticulitis, 2006–2011.

Year	Raw ED visits (N)	Raw admitted (n)	Weighted estimate of ED visits	Weighted admission rate for all patients (95% CI)*	Weighted admission rate for low-risk patients (95% CI)**
2006	50,636	28,392	238,248	55.7 (54.3, 57.2)	35.2 (33.5, 36.9)
2007	54,531	29,444	253,092	54.0 (52.6, 55,4)	32.5 (30.8, 34.2)
2008	59,191	30,968	257,257	51.9 (50.5, 53.3)	30.1 (28.7, 31.6)
2009	60,437	31,164	268,111	51.3 (50.0, 52.6)	30.0 (28.5, 31.5)
2010	62,231	31,660	280,398	50.6 (49.3, 51.9)	28.0 (26.7, 29.3)
2011	67,959	32,848	302,612	48.5 (47.2, 49.7)	26.8 (25.4, 28.2)
Average	59,164	30,744	266,620		

* p<0.001 trend,

** p<0.001 trend.

**Table 3:** National estimates of emergency department (ED) volume and surgery rate for diverticulitis, 2006–2011.

Year	National estimate of ED visits	In dataset (N)	Admitted (n)	Percentage rate of surgery for all patients (95% CI)	Percentage rate of surgery for admitted (95% CI)*	Percentage rate of surgery for all low-risk patients (95% CI)**	Percentage rate of surgery for admitted, low-risk patients (95%CI)***
2006	238,248	50,636	28,392	6.46 (6.15, 6.77)	11.72 (11.17, 12.27)	4.01 (3.59,4.41)	11.53 (10.40, 12.67)
2007	253,092	54,531	29,444	5.71 (5.42, 5.99)	10.73(10.18, 11.29)	3.59 (3.20,3.98)	11.31 (10.17, 12.47)
2008	257,257	59,191	30,968	5.51 (5.22,5.79)	10.792(10.25, 11.33)	3.00 (2.65, 3.36)	10.20 (9.00, 11.40)
2009	268,111	60,437	31,164	5.03 (4.78, 5.27)	9.98 (9.49, 10.47)	2.66 (2.33, 3.00)	9.12 (8.04, 10.21)
2010	280,398	62,231	31,660	4.99 (4.74, 5.24)	10.10 (9.56, 10.64)	2.40 (2.10, 2.70)	8.89 (7.76, 10.03)
2011	302,612	67,959	32,848	4.74 (4.51, 4.97)	10.00 (9.51, 10.49)	2.20 (1.91, 2.48)	8.51 (7.44, 9.57)
Average	266,620	59,164	30,744	5.36 (5.25, 5.47)	10.54(5.25, 5.47)	2.96 (2.82, 3.10)	10.01 (9.55, 10.48)

* p<0.001 trend,

** p<0.001 trend,

*** p<0.01 trend.

**Table 4:** Association of patient characteristics and emergency department (ED) admission rates for diverticulitis, 2011.

			OR (95% CI)	
			
	ED visits (N)	Admission rate (%)	Bivariate	Multivariate
Age				
10-year increase			1.22 (1.21, 1.23)	1.00 (0.98, 1.01)
Gender				
Male	29,222	45.8	Reference	Reference
Female	38,164	49.6	1.16 (1.12, 1.20)	0.84 (0.81, 0.88)
Insurance				
Medicare	23,541	56.6	1.73 (1.67, 1.80)	0.92 (0.87, 0.97)
Medicaid	5,631	45.7	1.04 (0.98, 1.11)	0.85 (0.79, 0.91)
Private insurance	29,355	43.7	Reference	Reference
Self-pay/no charge	6,672	39.5	0.87 (0.82, 0.92)	0.95 (0.89, 1.02)
Other	2,035	44.8	1.18 (1.07, 1.30)	1.15 (1.02, 1.29)
Income				
Lowest quartile	15,817	47.7	Reference	Reference
Second quartile	16,084	46.2	0.99 (0.94, 1.04)	1.03 (0.96, 1.09)
Third quartile	17,557	47.4	0.97 (0.92, 1.02)	1.05 (0.99, 1.12)
Highest quartile	16,620	50.6	0.98 (0.93, 1.05)	1.10 (1.02, 1.18)
