# Supplementary material for: Trends in Hospital Admission and Surgical Procedures Following ED visits for Diverticulitis
Source: West J Emerg Med. 2016 Jun 13;17(4):409–17. doi: 10.5811/westjem.2016.4.29757 (PMC4944797; doi:10.5811/westjem.2016.4.29757)
Supplement: Supplementary file 1 [file wjem-17-409-s001.docx]

**Appendix A. Principal Diagnoses for Which Diverticulitis was a Secondary Diagnosis**

| ICD9 and Description | N | % |
| --- | --- | --- |
| 78900: ABDOM PAIN NOS (Begin 1994) | 2,873 | 8.33 |
| 78904: ABDOM PAIN LLQ (Begin 1994) | 1,775 | 5.15 |
| 78909: ABDOM PAIN NEC (Begin 1994) | 1,420 | 4.12 |
| 5789 : GASTROINTEST HEMORR NOS | 436 | 1.26 |
| 78903: ABDOM PAIN RLQ (Begin 1994) | 380 | 1.10 |
| 56400: UNSPECIFIED CONSTIPATION (Begin 2001) | 293 | 0.85 |
| 78791: DIARRHEA (Begin 1995) | 275 | 0.80 |
| 78701: NAUSEA WITH VOMITING (Begin 1994) | 229 | 0.66 |
| 27651: DEHYDRATION (Begin 2005) | 212 | 0.61 |
| 78907: ABDOM PAIN GENERALIZED (Begin 1994) | 207 | 0.60 |
| 78906: ABDOM PAIN EPIGASTRIC (Begin 1994) | 196 | 0.57 |
| 33819: ACUTE PAIN NEC (Begin 2006) | 178 | 0.52 |
| 78902: ABDOM PAIN LUQ (Begin 1994) | 137 | 0.40 |
| 78901: ABDOM PAIN RUQ (Begin 1994) | 136 | 0.39 |
| 78060: FEVER NOS (Begin 2008) | 102 | 0.30 |
| 78703: VOMITING ALONE (Begin 1994) | 102 | 0.30 |
| 78702: NAUSEA ALONE (Begin 1994) | 78 | 0.23 |
| 78820: RETENTION OF URINE UNSPECIFED (Begin 1993) | 76 | 0.22 |
| 78905: ABDOM PAIN PERIUBILICAL (Begin 1994) | 66 | 0.19 |
| 56032: Fecal impaction (Begin 2010) | 65 | 0.19 |
| 56210: DVRTCLO COLON (W/O HMRHG) (Begin 1980) | 63 | 0.18 |
| 56212: DVRTCLO COLON W HMRHG (Begin 1991) | 41 | 0.12 |
| 56409: OTHER CONSTIPATION (Begin 2001) | 34 | 0.10 |
