# Supplementary material for: Trends in Hospital Admission and Surgical Procedures Following ED visits for Diverticulitis
Source: West J Emerg Med. 2016 Jun 13;17(4):409–17. doi: 10.5811/westjem.2016.4.29757 (PMC4944797; doi:10.5811/westjem.2016.4.29757)
Supplement: Supplementary file 2 [file wjem-17-409-s002.docx]

**Appendix B. ICD-9 codes excluded for critical illness and complicated diverticulitis.**

| ICD Code | Description |
| --- | --- |
| 5695 | INTESTINAL ABSCESS |
| V4589 | POSTSURGICAL STATES NEC |
| 56722 | PERITONEAL ABSCESS |
| 5180 | PULMONARY COLLAPSE |
| 5679 | PERITONITIS NOS |
| 0389 | SEPTICEMIA NOS |
| 9974 | SURG COMPLIC-GI TRACT |
| 5609 | INTESTINAL OBSTRUCT NOS |
| 56721 | PERITONITIS (ACUTE) GEN |
| 51881 | RESPIRATORY FAILURE |
| 5693 | RECTAL & ANAL HEMORRHAGE |
| 56089 | INTESTINAL OBSTRUCT NEC |
| 5961 | INTESTINOVESICAL FISTULA |
| 5781 | MELENA |
