# Supplementary material for: Trends in Hospital Admission and Surgical Procedures Following ED visits for Diverticulitis
Source: West J Emerg Med. 2016 Jun 13;17(4):409–17. doi: 10.5811/westjem.2016.4.29757 (PMC4944797; doi:10.5811/westjem.2016.4.29757)
Supplement: Supplementary file 3 [file wjem-17-409-s003.docx]

**Appendix C. ICD-9 codes used in risk adjustment in addition to Elixhauser Conditions.**

| ICD Code | Description |
| --- | --- |
| 276.51 | DEHYDRATION (Begin 2005) |
| 288.60 | LEUKOCYTOSIS NOS (Begin 2006) |
| 787.91 | DIARRHEA (Begin 1995) |
| 584.9 | ACUTE RENAL FAILURE NOS |
| 558.9 | NONINF GASTROENTERIT NEC |
| 780.60 | FEVER NOS (Begin 2008) |
| 458.9 | HYPOTENSION NOS |
| 263.9 | PROTEIN-CAL MALNUTR NOS |
| 486.0 | PNEUMONIA- ORGANISM NOS |
| 276.2 | ACIDOSIS |
| 785.0 | TACHYCARDIA NOS |
| 008.45 | CLOSTRIDIUM DIFF |
| 787.01-3 | VOMITING |
