# Supplementary material for: Trends in Hospital Admission and Surgical Procedures Following ED visits for Diverticulitis
Source: West J Emerg Med. 2016 Jun 13;17(4):409–17. doi: 10.5811/westjem.2016.4.29757 (PMC4944797; doi:10.5811/westjem.2016.4.29757)
Supplement: Supplementary file 4 [file wjem-17-409-s004.docx]

| **Appendix D. Admission Rates & Odds Ratios for Elixhauser Conditions***   \|  \| ED visits \| Admission Rate \| OR (95% CI) \| \| \| --- \| --- \| --- \| --- \| --- \| \| (N) \| ( %) \| Bivariate \| Multivariate \| \| Valvular disease \| 1,116 \| 81.4 \| 5.04 (4.30, 5.89) \| 2.30 (1.91, 2.76) \| \| Pulmonary circulation disease \| 319 \| 83.4 \| 6.60 (4.86, 8.97) \| 1.59 (1.09, 2.31) \| \| Peripheral vascular disease \| 1,611 \| 74.8 \| 3.78 (3.35, 4.25) \| 1.80 (1.56, 2.08) \| \| Arterial hypertension, complicated \| 26,963 \| 62 \| 2.76 (2.66, 2.85) \| 1.74 (1.66, 1.82) \| \| Paralysis \| 165 \| 84.8 \| 7.26 (4.66, 11.31) \| 4.15 (2.49, 6.93) \| \| Other neurologic disease \| 1,521 \| 77.8 \| 4.30 (3.78, 4.88) \| 2.59 (2.23, 3.01) \| \| Chronic pulmonary disease \| 6,604 \| 71.5 \| 3.22 (3.04, 3.42) \| 2.05 (1.91, 2.20) \| \| Diabetes \| 8,536 \| 61 \| 1.86 (1.77, 1.95) \| 1.03 (0.97, 1.10) \| \| Diabetes, complicated \| 669 \| 78 \| 5.16 (4.24, 6.27) \| 1.57 (1.23, 2.01) \| \| Hypothyroidism \| 5,118 \| 75.8 \| 3.93 (3.67, 4.21) \| 2.77 (2.55, 3.00) \| \| Renal failure \| 2,428 \| 82.7 \| 6.47 (5.79, 7.23) \| 2.03 (1.77, 2.33) \| \| Liver diseases \| 1,489 \| 70.3 \| 2.68 (2.38, 3.02) \| 1.63 (1.42, 1.88) \| \| AIDS/HIV \| 35 \| 74.3 \| 3.37 (1.52, 7.46) \| 1.64 (0.62, 4.35) \| \| Lymphoma \| 196 \| 79.6 \| 4.10 (2.86, 5.90) \| 2.38 (1.55, 3.65) \| \| Metastatic cancer \| 264 \| 87.9 \| 8.26 (5.66, 12.06) \| 5.45 (3.53, 8.40) \| \| Solid tumor without metastasis \| 504 \| 74 \| 3.59 (2.91, 4.42) \| 2.41 (1.87, 3.10) \| \| Rheumatoid arthritis/collagen vascular diseases \| 1,190 \| 79.3 \| 4.58 (3.96, 5.31) \| 2.99 (2.51, 3.56) \| \| Coagulopathy \| 721 \| 89.7 \| 9.98 (7.80, 12.77) \| 3.79 (2.86, 5.02) \| \| Obesity \| 4,686 \| 84.1 \| 7.85 (7.21, 8.55) \| 6.53 (5.92, 7.20) \| \| Weight loss \| 755 \| 90.7 \| 13.19 (10.24, 16.99) \| 3.95 (2.90, 5.38) \| \| Fluid and electrolyte disorders \| 8,664 \| 86.8 \| 10.87 (10.15, 11.64) \| 5.94 (5.48, 6.45) \| \| Blood loss anemia \| 137 \| 94.9 \| 19.38 (9.00, 41.76) \| 11.32 (4.87, 26.30) \| \| Deficiency anemia \| 4,731 \| 89.5 \| 11.54 (10.47, 12.72) \| 5.67 (5.08, 6.33) \| \| Alcohol abuse \| 851 \| 82.8 \| 6.10 (5.07, 7.35) \| 4.42 (3.58, 5.46) \| \| Drug abuse \| 542 \| 80.1 \| 5.28 (4.23, 6.58) \| 4.27 (3.29, 5.53) \| \| Psychoses \| 1,025 \| 74.7 \| 3.72 (3.21, 4.32) \| 2.52 (2.11, 3.00) \| \| Depression \| 3,993 \| 77.5 \| 4.48 (4.13, 4.85) \| 3.25 (2.97, 3.57) \| \| Risk factor \| 5,020 \| 39.6 \| 0.70 (0.66, 0.75) \| 0.55 (0.51, 0.59) \| \| GI disease \| 2,123 \| 82.5 \| 5.75 (5.12, 6.47) \| 2.94 (2.56, 3.39) \| \| Disease severity \| 6,610 \| 75.9 \| 4.33 (4.06, 4.60) \| 1.73 (1.59, 1.87) \| |
| --- | --- | --- | --- | --- | --- | --- | --- | --- | --- | --- | --- | --- | --- | --- | --- | --- | --- | --- | --- | --- | --- | --- | --- | --- | --- | --- | --- | --- | --- | --- | --- | --- | --- | --- | --- | --- | --- | --- | --- | --- | --- | --- | --- | --- | --- | --- | --- | --- | --- | --- | --- | --- | --- | --- | --- | --- | --- | --- | --- | --- | --- | --- | --- | --- | --- | --- | --- | --- | --- | --- | --- | --- | --- | --- | --- | --- | --- | --- | --- | --- | --- | --- | --- | --- | --- | --- | --- | --- | --- | --- | --- | --- | --- | --- | --- | --- | --- | --- | --- | --- | --- | --- | --- | --- | --- | --- | --- | --- | --- | --- | --- | --- | --- | --- | --- | --- | --- | --- | --- | --- | --- | --- | --- | --- | --- | --- | --- | --- | --- | --- | --- | --- | --- | --- | --- | --- | --- | --- | --- | --- | --- | --- | --- | --- | --- | --- | --- | --- | --- | --- | --- | --- | --- | --- | --- | --- | --- | --- | --- |

*In our analysis there were only three visits for diverticulitis who had peptic ulcer as a comorbidity which were all admitted, making that OR unreportable
